# Supplementary material for: Leptin-Induced HLA-G Inhibits Myometrial Contraction and Differentiation
Source: Cells. 2022 Mar 10;11(6):954. doi: 10.3390/cells11060954 (PMC8946078; doi:10.3390/cells11060954)
Supplement: Supplementary file 1 [file cells-11-00954-s001.zip › cells-1600880-supplementary.pdf]

Figure S1 - Supplemental (Wendremaire *et al.*)

(A)

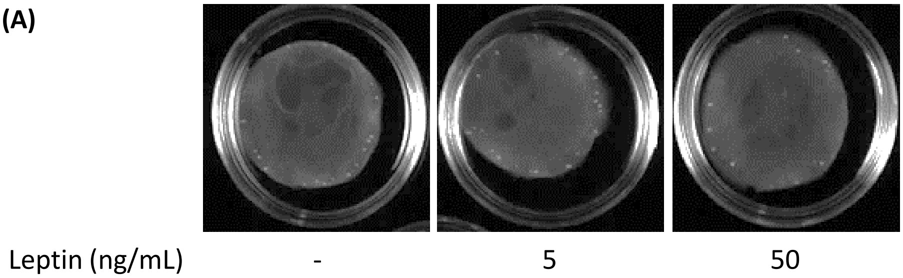

(B)

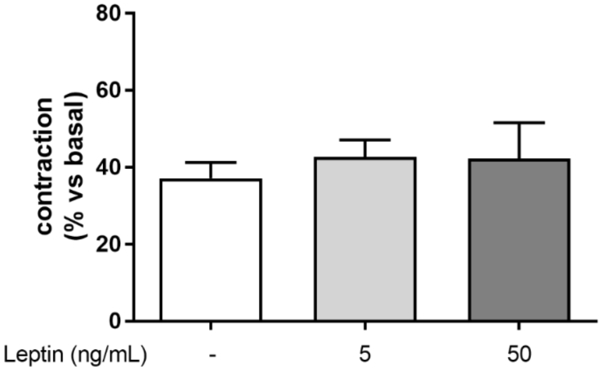

Supplemental Figure S1: Leptin-induced contraction in myometrial cells. Myocytes were stimulated or not with leptin at 5 or 50 ng/mL. **(A)** Photographs of collagen lattices of myometrial cells in presence or absence of leptin, for 96 h. Images shown are representative of five experiments. **(B)** Mean percentage contraction of collagen lattices ± SEM after 96 h of stimulation with leptin, compared to the basal surface area, n=5.

Figure S2 - Supplemental (Wendremaire *et al.*)

(A)

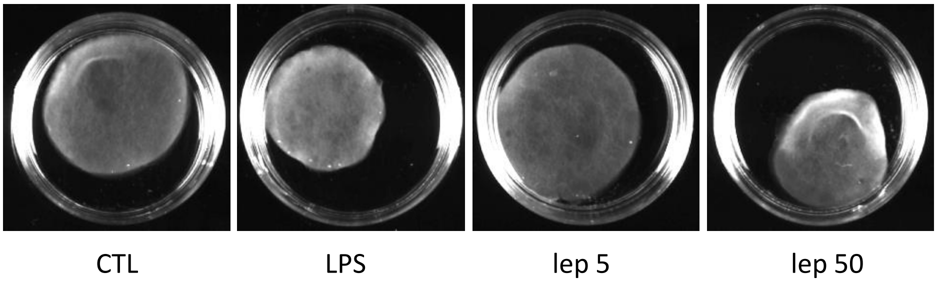

(B)

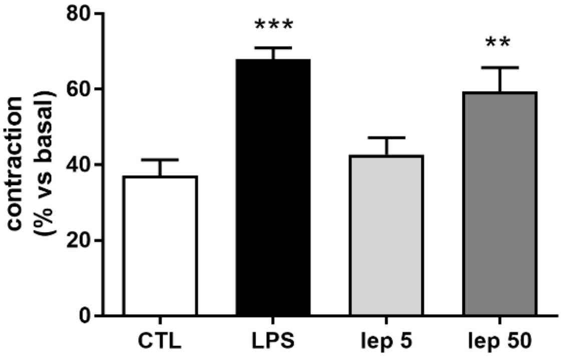

Supplemental Figure S2: Leptin- and LPS-induced myometrial contraction on myocytes/macrophages co-culture. Myometrial cells and macrophages were stimulated with LPS or leptin at 5 and 50 ng/mL. **(A)** Photographs of collagen lattices of co-cultures of myocytes and macrophages in presence or absence of LPS or leptin, for 96 h. Images shown are representative of five experiments. **(B)** Mean percentage contraction of collagen lattices ± SEM after 96 h of stimulation with LPS or leptin, compared to the basal surface area, n=5. **$p<0.01$ and ***$p<0.001$ *versus* CTL.

Figure S3 - Supplemental (Wendremaire *et al.*)

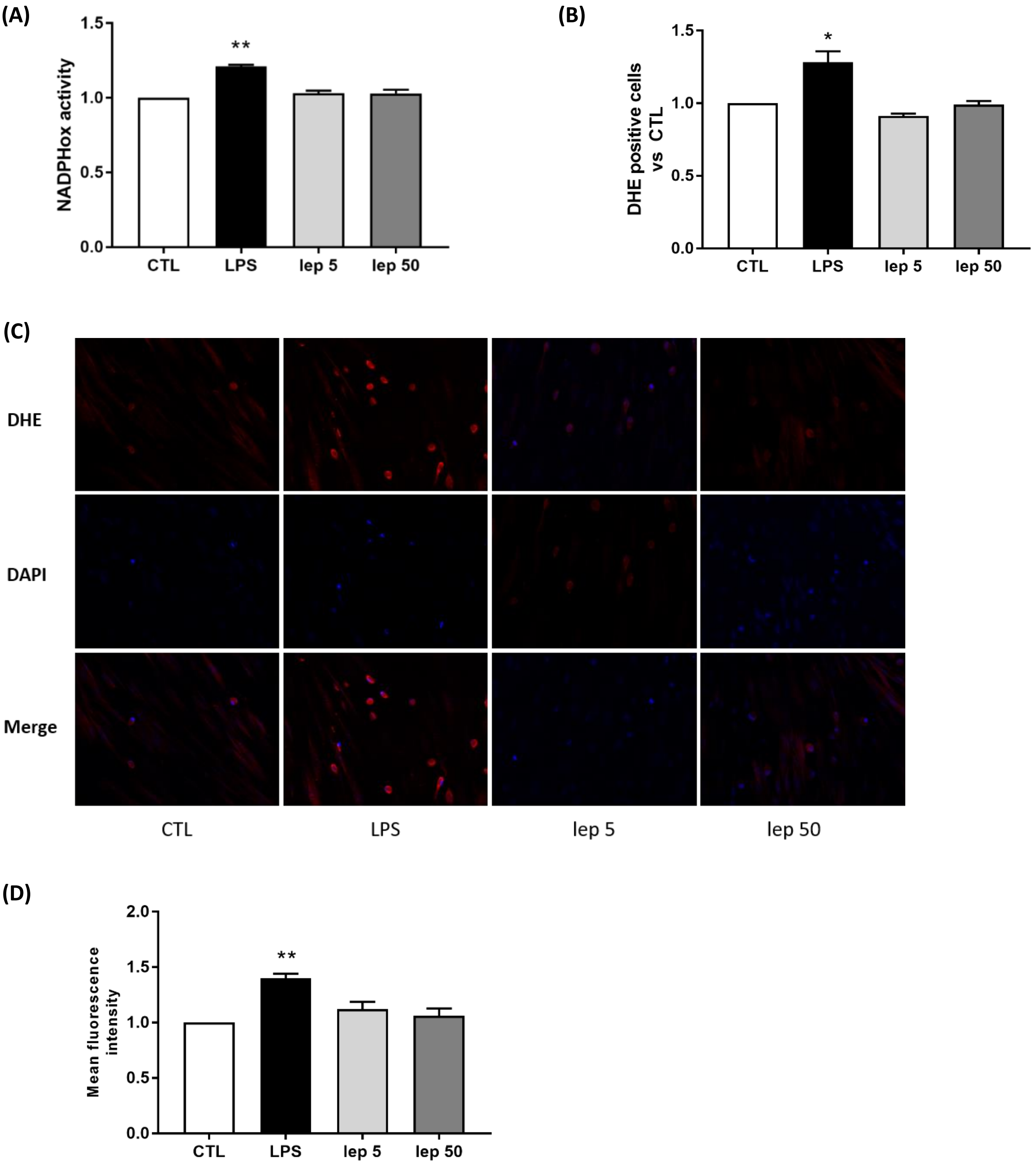

Supplemental Figure S3: Effect of leptin on ROS production in co-cultured cells. Myometrial cells and macrophages were stimulated with LPS or leptin at 5 and 50 ng/mL. **(A)** NADPHox activity measured by luminometric analysis and related to the protein levels. Relative values *versus* CTL are represented as mean ± SEM, n=4, **p<0.01 *versus* CTL. **(B)** Proportions of DHE-positive cells represented as fold induction *versus* CTL (mean ± SEM), n=4, *p<0.05 *versus* CTL. **(C)** Fluorescence images of DHE (red) and DAPI (blue) staining, taken with an epifluorescence microscope at x200 magnification in random chosen fields, representative of five pictures taken for each condition, n=5. **(D)** Mean fluorescent intensity of DHE staining represented as fold induction *versus* CTL (mean ± SEM), n=5, **p<0.01 *versus* CTL.

Figure S4 - Supplemental (Wendremaire *et al.*)

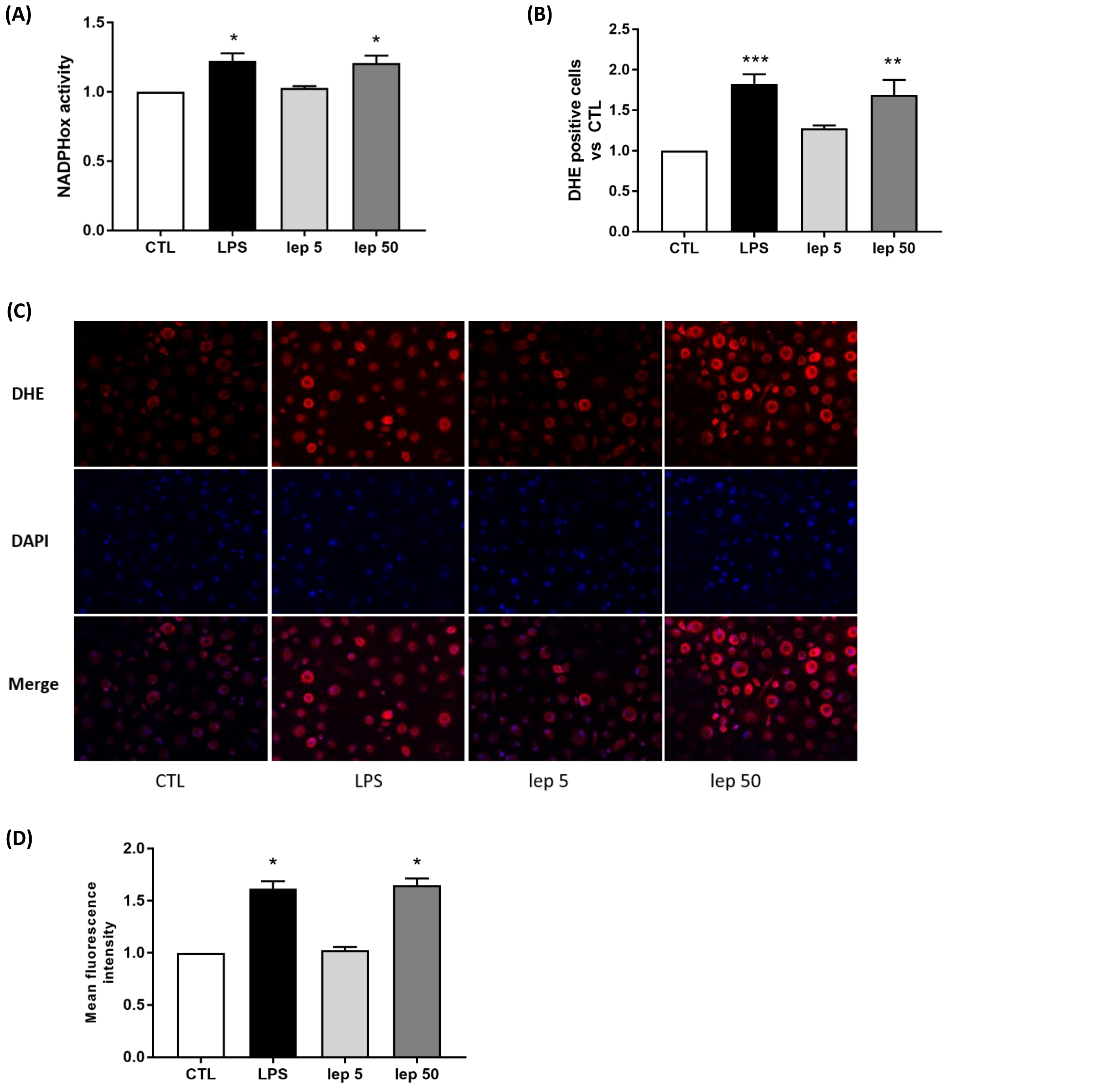

Supplemental Figure S4: Effect of leptin on ROS production in macrophages. Macrophages were stimulated with LPS or leptin at 5 and 50 ng/mL. **(A)** NADPHox activity measured by luminometric analysis and related to the protein levels. Relative values *versus* CTL are represented as mean ± SEM, n=8, *p<0.05 *versus* CTL. **(B)** Proportions of DHE-positive cells represented as fold induction *versus* CTL (mean ± SEM), n=5, **p<0.01 and ***p<0.001 *versus* CTL. **(C)** Fluorescence images of DHE (red) and DAPI (blue) staining, taken with an epifluorescence microscope at x200 magnification in random chosen fields, representative of five pictures taken for each condition, n=4. **(D)** Mean fluorescent intensity of DHE staining represented as fold induction *versus* CTL (mean ± SEM), n=4, *p<0.05 *versus* CTL.
